# Motor neuron axonal excitability changes in the clinical course of amyotrophic lateral sclerosis

**DOI:** 10.1007/s10072-025-08443-w

**Published:** 2025-08-25

**Authors:** Panagiotis Kokotis, Eleni Bakola, Martin Schmelz, Michalis Rentzos, Georgia Papagiannopoupou, Georgios Tsivgoulis

**Affiliations:** 1 First Department of Neurology, School of Medicine, National and Kapodistrian University of Athens, “Aigineition” Hospital, Athens, Greece; 2 Second Department of Neurology, School of Medicine, National and Kapodistrian University of Athens, “Attikon” Hospital, Athens, Greece; 3 Mannheim Medical School, Department of Anesthesiology Ethics approval, University of Heidelberg, Mannheim, Germany

## Abstract

Amyotrophic lateral sclerosis (ALS) is a fatal disease characterized by slowly progressive degeneration of upper motor neurons (UMNs) and lower motor neurons (LMNs). Although the pathogenesis of sporadic form of ALS has not been fully elucidated, the initial damage mostly leads to hyperexcitability of the central and peripheral motor neuron. The results of our study aimed to confirm the changes in the excitability of the peripheral motor axon and contribute to the emergence of these measurements as potential biomarkers for disease progression. A total of 56 ALS patients [24 women (43%), median age 62.5 (interquartile range: 53.75–70.25) years] were finally included in the study. Twenty-four healthy controls that were age- and sex-matched to the cases [12 women (50%), mean age 58.46 ± 8.84] were recruited. The first main finding of our study is the fact that abnormalities of the voltage gated K^+^ ion channels were constantly present in ALS patients than in the controls. The multivariate analysis revealed that Superexcitability 7ms lower than − 21.06%, related to shorter survival. Additionally using receiver operating characteristic (ROC) curves, the c-statistic showed Superexcitability 7ms moderate predictive ability. The clinical significance of our results is that Superexcitability 7ms can be used as a biomarker not only for survival but also for disease progression.

**Supplementary Information:**

The online version contains supplementary material available at 10.1007/s10072-025-08443-w.

## Introduction

ALS is a devastative neurologic disease characterized by progressive degeneration of both UMNs and LMNs, leading to muscle weakness and atrophy [1]. In approximately 5–10% of cases a hereditary component is evident although most ALS patients arise sporadically.

Although the pathogenesis of sporadic form of ALS has not been fully elucidated, the initial damage mostly leads to hyperexcitability of UMNs and LMNs [2]. Neuronal hyperexcitability appears to be the initial process also in other progressive neurodegenerative diseases such as Alzheimer’s disease (AD) [3]. In AD the intracellular increase of Ca2 + and glutamate results in neuron destruction, like in ALS. Specifically, in ALS, abnormalities in the conductance of membrane Na + and K + voltage-gated channels contribute equally to the development of motor axon membrane hyperexcitability, thus leading to the generation of symptoms such as muscle cramps and fasciculations. Furthermore, they promote a neurodegenerative cascade through Ca2+-mediated processes, leading to cell death [4].

For more than two decades, tracking threshold techniques have been developed and applied for the in vivo examination and monitoring of motor neuron excitability, both central and peripheral. Multiple excitability measurements (MEM) protocol is used specifically to monitor changes of the peripheral motor neuron threshold, testing parameters such as strength duration time constant (SDTC), electrotonus and recovery cycle [5]. Since then, several studies have been published using the above parameters as biomarkers for the progression of ALS. Some of them even attempt to correlate the changes of the MEM with the survival of ALS patients [6].

However, apart from the prognostic value, MEM have recently gained importance, after the developments in the therapeutic approach of the disease. The initial changes in the excitability of these membrane voltage-gated Na + and K + channels, have become one of the therapeutic targets of the disease. Modulation of these channels function in ALS indeed, has led to significant symptomatic improvement accompanied by stabilization of MEM [4]. Additionally, axonal ion channel dysfunction appears to develop with disease progression and correlates with survival, thus serving as a potential therapeutic biomarker in ALS.

Thus, it seems to be justified by the attempt of many studies to link the MEM of the central and peripheral motor neuron with survival in ALS. Of these, very few have combined measurements of both UMNs and LMNs excitability simultaneously, showing advantages of the peripheral MEM to correlate with survival in ALS patients [7].

A recent meta-analysis of studies with the MEM protocol in ALS patients showed that the majority of the original 25 studies, included in the analysis, were significantly biased mainly for reasons of patient selection (not consecutive) and the low amplitude of the compound motor evoked potential (CMAP) of the median nerve, which affects the reliability of the results. Consequently, their measurements cannot be used as potential biomarkers in ALS, except in 4 of them [8]. Thus, new prospective studies, with diagnostic tests accuracy, are reasonable to firmly establish the utility of the MEM in individuals suspected of an ALS diagnosis.

In view of the former considerations, we sought to conduct a prospective study using the MEM in consecutive ALS patients, as early as possible after the onset of the disease. The results of our study aimed to confirm the changes in the excitability of the peripheral motor axon and contribute to the emergence of these measurements as potential biomarkers for disease progression.

## Materials and methods

Consecutive patients fulfilling the El Escorial criteria for possible or probable ALS (World Federation of Neurology, 1994) were evaluated very early after the diagnosis of the disease, at the Laboratory of Clinical Neurophysiology in the First Department of Neurology of National & Kapodistrian University of Athens. We excluded coincidental carpal tunnel syndrome based on clinical examination and nerve conduction studies. We also excluded ALS patients with other neurological disorders possibly interfering with motor neuron damage (diabetic neuropathy, cervical spondylosis, alcohol abuse and history of poliomyelitis). A total of 56 ALS patients [24 women (43%), median age 62.5 (interquartile range: 53.75–70.25 years] were finally included in the study. Their median age at onset was 61.50 (interquartile range: 52.00–70.00 years), and the median disease duration was 12 months (interquartile range: 6–18).

Twenty-four healthy controls that were age- and sex-matched to the cases [12 women (50%), mean age 58.46 ± 8.84] were also recruited. Informed consent according to the Declaration of Helsinki was obtained from all participants and the study was approved by the local ethics committee.

### Standard nerve conduction studies (NCS)

The electrophysiological studies for all the patients were carried out in the Laboratory of Clinical Neurophysiology at the First Department of Neurology in the National & Kapodistrian University of Athens, in a controlled temperature room using standardized methods. Compound motor action potentials (CMAPs) were recorded from the abductor pollicis brevis (APB) muscle, and amplitudes peak to peak of the CMAPs were estimated for the median nerve, using surface electrodes.

## Multiple excitability measurements

Multiple excitability properties were measured in all patients and controls for the median nerve at the wrist and recorded from APB muscle, (QTRAC with multiple protocol TROND, Institute of Neurology, London, UK), as reported elsewhere (9). The skin temperature near the stimulus site was maintained above 32 °C. The following excitability indices were measured and included: strength-duration time constant (SDTC), threshold electrotonus (TE), refractoriness, superexcitability, and late subexcitability of the recovery cycle of axonal excitability with a single supramaximal conditioning stimulus and current-threshold relationship. SDTC was calculated from the relationship between stimulus intensity and duration to evoke a target potential, using the formulation of Weiss (10). In TE, a 100ms sub-threshold polarizing pulse was delivered as a conditioning stimulus, and the threshold change to produce a target CMAP response (40% maximus) was measured. The recovery cycle was recorded as the recovery of axonal membrane excitability following a supramaximal conditioning stimulus. A current-threshold relationship was obtained by tracking threshold changes, following a sub-threshold 200 ms polarizing current.

### Statistical analysis

Categorical variables are presented as the number of patients with the corresponding percentages. Continuous variables are presented as mean ± standard deviation (SD, normal distribution) or as median with interquartile range (IQR, skewed distribution). Statistical comparisons between categorical variables were performed using χ2 test, or in case of small expected frequencies, Fisher’s exact test. Continuous variables were compared using the unpaired t-test or Mann–Whitney U test, as indicated.

Overall survival was defined as the time of the evaluation until death. For time-to-event outcomes, the lengths of time to a first event were compared using the log-rank test, while the Kaplan-Meier method was used to estimate the absolute risk of each event for each group, using the Cox proportional hazard model. For excitability indices, the cut-off values were determined as the mean values of the control subjects, and patients with ALS were divided into two subgroups with a higher and lower value than the normal mean of each excitability index.

To identify prognostic factors associated with survival, multivariate analysis was performed using the Cox proportional hazard model and HRs with 95% CΙs were estimated. A cutoff of *p* < 0.05 was used to select variables for inclusion in multivariable analyses. Furthermore, age and type of disease onset were included a priori in the model, as they are recognized as important variables influencing survival.

Finally, a receiver operating characteristic (ROC) curve with corresponding area under the curve (AUC) and 95% CIs, was generated to estimate the diagnostic utility of motor axonal excitability indices that after multivariate analysis remained independently associated with survival in ALS patients.

A probability level of *p* < 0.05 was chosen for significance. All statistical analyses were performed using the R software version 3.5.0.

## Results

The clinical state for most of the patients was recorded up to 7 years after the diagnosis of the disease. In 37.5% of the patients, the disease started with bulbar symptoms, and 32 deaths were reported during the follow-up period.

ALS patients manifested higher Refractoriness 2ms (*p* = 0.031), TEd (90-100ms) (*p* < 0.001), and lower Superexcitability 7ms (*p* = 0.013), Subexcitability (*p* = 0.006), and CMAP (*p* < 0.001) than the healthy controls. The multiple excitability measurements result of the included ALS-patients and controls are summarized in Supplement file Table 1.

## Univariate analysis

The results of univariate Cox regression analysis are shown in Table 1 (only variables with *p* < 0.05 are included). Female gender and disease duration > 12months were associated with shorter survival (*p* = 0.0422 and *p* < 0.001, respectively). Superexcitability 7ms lower than − 21.06% (i.e. smaller indices, being less negative) and TEd (10-20ms) higher than 68.90% were also related to shorter survival (*p* < 0.001 and *p* = 0.004, respectively).

**Table 1:** Univariate Cox regression analysis of prognostic factors in amyotrophic lateral sclerosis (only variables with *p*< 0.05 are included in the table)

Variables	HR (95% CI)	*p*-value*
Gender		
Women	1.00	
Men	0.4548 (0.21–0.99)	**0.042**
Disease duration**		
> 12mo	1.00	
< 12mo	0.18 (0.07–0.42)	**< 0.001**
Superexcitability 7ms**		
> −21.06%	1.00	
< −21.06%	5.85 (2.21–15.52)	**< 0.001**
TEd (10-20ms)**		
> 68.90%	1.00	
< 68.90%	0.31 (0.15–0.67)	**0.004**

*p values were calculated by log-rank test.

** cut-off values were determined as the mean value of normal controls.

The female patients appeared with disease duration longer (> 12 months) than men: 14/24 vs. 9/32 (Fisher’s exact test *p* = 0.02).

Simultaneously the patients with more negative superexcitability 7ms (<−21.06%) had higher TEd 10–20 ms (> 68.90%), than patients with less negative: 20/26 vs. 13/29 (Fisher’s exact test *p* = 0.01).

### Kaplan-Meier plots of survival probabilities

**Fig. 1:**
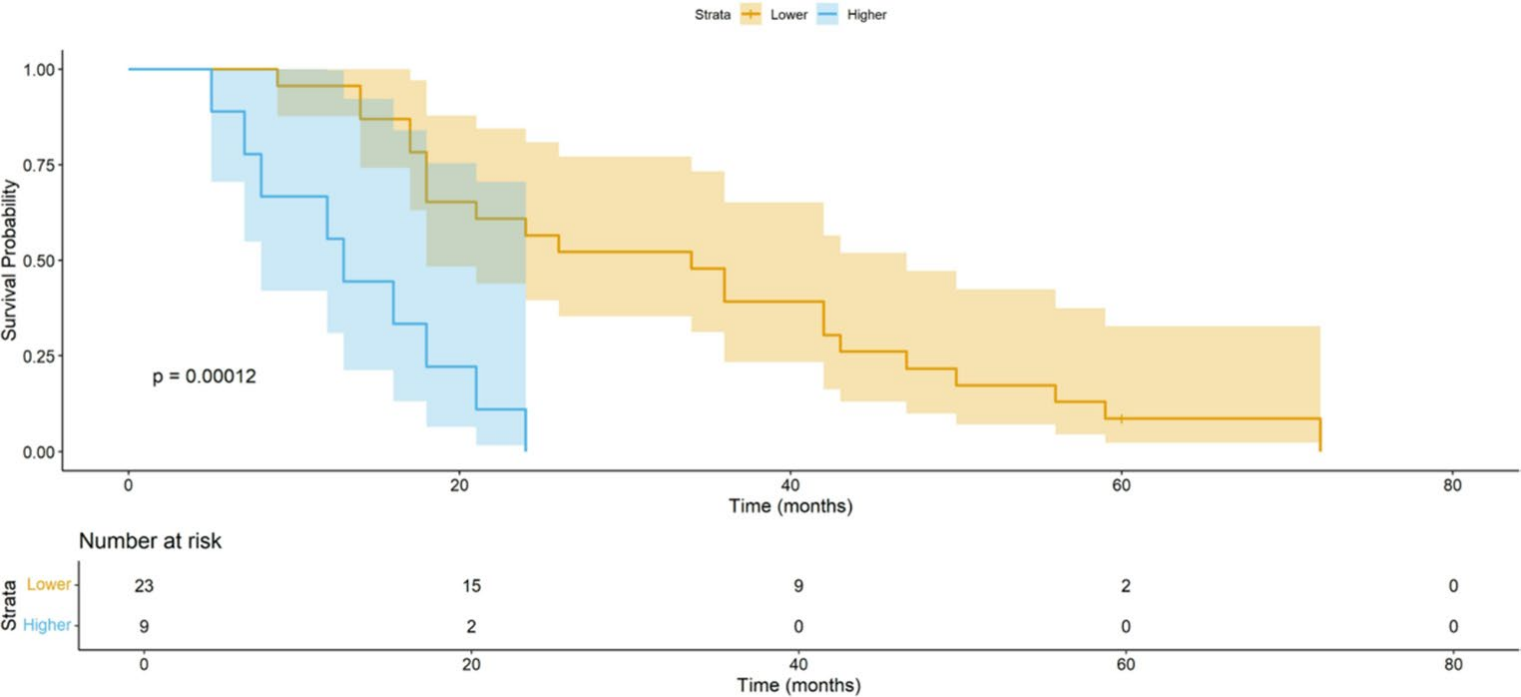
Kaplan-Meier plots of survival probabilities according to **Superexcitability 7ms**. Patients were divided into two groups according to higher and lower than normal mean value. Higher **Superexcitability** 7ms was associated with shorter survival

**Fig. 2:**
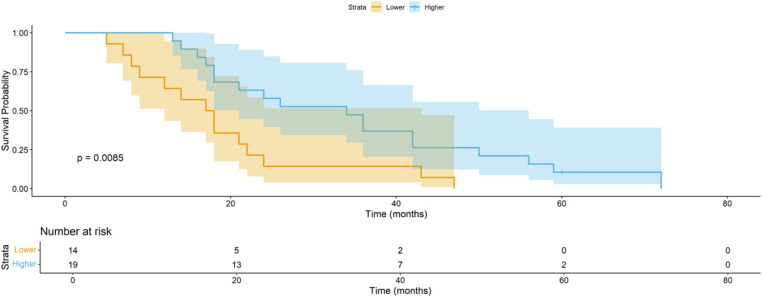
Kaplan-Meier plots of survival probabilities according to **T2D (10-20ms).** Patients were divided into two groups according to higher and lower than normal mean value. Lower T2D (10-20ms) was associated with shorter survival

Figures 1 and 2 show Kaplan-Meier curves for survival of the patient subgroups divided into two groups according to higher and lower than normal mean value of Superexcitability 7ms and TEd (10-20ms).

## Multivariate analyses

Cox proportional hazard model including the variables with a cutoff of *p* < 0.05 in univariate analysis [i.e. gender, disease duration, Superexcitability 7ms and TEd (10-20ms)] and age and type of disease onset is depicted in Table 2 (model 1). Superexcitability 7ms lower than − 21.06% was related to shorter survival (adjusted HR 4.66; 95%CI: 1.37–15.88; *p* = 0.014), whereas all the other parameters including gender, disease duration and TEd (10-20ms) had no significant association with survival when adjusted with other factors. In order to strengthen our statistical analysis, we repeated the multivariate analyses after including the variable of CMAP, which is well known prognostic factor associated with survival in ALS patients. These analyses documented similar results, as shown in Table 3 (model 2).

**Table 2:** Multivariate analysis of prognostic factors in amyotrophic lateral sclerosis (model 1)

Variables	Adjusted HR (95% CI)	*p*-value*
Gender		
Women	1.00	
Men	0.61 (0.19–1.95)	0.408
Disease duration**		
> 12mo	1.00	
< 12mo	0.63 (0.23–1.72)	0.366
Age	1.01(0.98–1.04)	0.678
Type of disease onset		
Bulbar	1.00	
Limb	0.71 (0.25–2.05)	0.531
Superx 7ms**		
> −21.06%	1.00	
< −21.06%	4.26 (1.18–15.32)	**0.027**
T2D (10-20ms)**		
> 68.90%	1.00	
< 68.90%	1.00 (0.34–2.98)	0.993

* p values were calculated by log-rank test

** cut-off values were determined as the mean value of normal controls

**Table 3:** Multivariate analysis of prognostic factors in amyotrophic lateral sclerosis (model 2)

Variables	Adjusted HR (95% CI)	*p*-value*
Age	1.01 (0.97–1.04)	0.680
CMAP	1.00 (0.88–1.13)	0.956
Gender		
Women	1.00	
Men	0.61 (0.19–1.99)	0.411
Disease duration**		
> 12mo	1.00	
< 12mo	0.62 (0.21–1.86)	0.395
Type of disease onset		
Bulbar	1.00	
Limb	0.72 (0.25–2.04)	0.532
Superx 7ms**		
> −21.06%	1.00	
< −21.06%	4.26 (1.18–15.41)	**0.027**
T2D (10-20ms)**		
> 68.90%	1.00	
< 68.90%	1.02 (0.32–3.22)	0.979

* p values were calculated by log-rank test

** cut-off values were determined as the mean value of normal controls

## ROC curves

Finally, receiver operating characteristic (ROC) curves were generated to estimate the diagnostic utility of CMAP and Superexcitability 7ms, the excitability measure that after multivariate analyses remained independently associated with survival in ALS patients. The c-statistic showed no predictive ability of CMAP (AUC: 0.489, 95%CI: 0.285–0.693) and moderate predictive ability of Superexcitability 7ms (AUC: 0.677, 95%CI: 0.480–0.874) (Fig. 3).

## Discussion

The clinical features of ALS vary greatly, depending on the extent of upper and lower motor neuron involvement, the initial level the symptoms of the disease appear, and how they spread. This heterogeneity of ALS and the absence of established biomarkers often make diagnosis difficult or delayed, as well as limiting prognosis, disease monitoring and therapeutic approach to the individual patient.

Responding to the need for reliable electrophysiological biomarkers, we conducted this prospective study, using careful and precise methods. We observed the consecutive of recruitment, did not include patients with other possible causes of peripheral motor neuron damage, compared the measurement results with an age matched control group, and examined the patients soon after diagnosis and as early as possible from the onset of symptoms, which was determined by accuracy. As a result of the above, the patients who were finally included in the study had a high enough CMAP amplitude (with 75% of them being above the normal limits) which ensures that we did not have advanced atrophy in the APB that would affect both the quality of the recording and the results of the measurements.

The first main finding of our study is the fact that abnormalities of the voltage gated K^+^ ion channels were constantly present in ALS patients than in the controls. They are reflected by significantly increased threshold depolarizing electrotonus, recovery cycle’s higher Superexcitability and lower Subexcitability, all indices of juxtra-paranodal potassium channels decreased current. The above changes of potassium channels lead to depolarization of the motor neuron axon membrane. These findings are in line with several previous studies [6, 7].

Additionally, our study revealed a significant increase in Refractoriness. The last measurement is usually increased due to membrane depolarization which delays the sodium channels inactivation. In our case the depolarization could attribute to the potassium channels changes, although refractoriness is very sensitive to other factors like temperature changes, or the ability of neuromuscular junction to transmit action potentials faithfully, which in ALS it is an issue [5].

We did not find significant prolongation of strength duration time constant (SDTC) as other studies revealed in ALS patients, ascribed to upregulation of persistent Na + conductance. The above increase of SDTC was most prominent in ALS patients with a moderate degree of lower motor neuron dysfunction [6, 7], while our patients had minimum APB muscle atrophy as they were recruited enough early after the initiation of the symptoms.

The results of univariate Cox regression analysis of the patients’ base line characteristics revealed that female gender and disease duration > 12months were associated with shorter survival. The association of the longer disease duration with shorter survival, although it is reasonable, it depends on the median value which is used for the analysis. A recent study with longer than our mean disease duration (16.8 vs. 12 months) revealed exactly the opposite result [7]. The above imposes the significance of our study with enrollment of patients early in the progress of the disease.

The MEM univariate Cox regression analysis revealed that Surexcitability 7ms lower than − 21.06% (i.e. smaller indices, being less negative) and TEd (10-20ms) higher than 68.90% were also related to shorter survival (*p* < 0.001 and *p* = 0.004, respectively). Exploring their relationship, we found the above measurements to be significantly correlated, probably because they share the same underlying electrophysiologic process which is the reduction of the fast paranodal potassium channels current in ALS patients [11]. Thus, the multivariate analysis results unveiled the strong correlation of Superexcitability 7ms with survival, while Ted10-20 lost it.

According to our results, only Superexcitability 7ms, between all MEM, can be used as a prognostic factor while from conventional NCS the amplitude of the ABP CMAP cannot. And in fact, while on average the Superexcitability 7ms is significantly more negative than the controls, however, the less negative it is than the median value of the controls, the shorter the survival seems to be. This means that Superexcitability 7ms changes during the disease with a significant paranodal potassium channels current reduction in the early stages of the disease leading to a hyperexcitability of the motor neuron and the aforementioned cascade that ultimately leads to degeneration of the neuron. As the disease progresses there is a switch towards increased current through the potassium channels, reflected by the less negative value of Superexcitability 7ms. The later could be attributed to the depolarization of the neuronal membrane that occurs in the final stage of the disease before the death of the neuron [12]. Another recent theory is that during the disease progress, the initial hyperexcitability gives way to a hypoerexcitability of the motor neuron as a counterbalancing mechanism just before cell death [13].

The dysregulation of ion channels in ALS has been studied also by other researchers, but without agreement in the results, possibly due to the great heterogeneity in the basic characteristics of the patients included in each study and of the disease itself. A very recent one compares cortical and peripheral neuron hyperexcitability and finds a superiority of the peripheral motor neuron hyperexcitability factors as prognostic markers in ALS. Their patients had a longer duration of the disease and a much lower CMAP amplitude than ours, which means that they were in a more advanced stage of the disease. Presumably for this reason, they found only sodium channel-related SDTC and not the potassium channel-related variables as a predictor of survival. Because, as they conclude, at the early stages of the disease the motor neuron Superexcitability is due to the decrease of the current in the potassium channels and then to the increase of the current in the sodium channels [7]. Exactly the opposite are the results of an earlier study with the authors concluding that first, persistent Na + conductance increases, possibly associated with collateral sprouting, and then K + conductance declines [11].

However, the few reliable studies (not biased) using survival analysis for the ALS patients [8], instead of regression analyses [14], agree with our findings that superexcitability of the recovery cycle, can be used as a biomarker for the prognosis and monitoring of the disease, as it changes during its progress, such as the Kanai et al., study [6]. The crucial difference is that, in their study, superexcitability measurements lose their significance with survival in multivariate analysis, contrary to SDTC. This difference could be attributed to the longer range of disease duration in their patients (up to 68 months). Most researchers agree that the increase in SDTC due to increased current in Na + channels occurs in an advanced stage of the disease [4]. Nevertheless, Kanai et al. study [6] also supports that in the early phase of the disease, potassium conductance primarily decreases, the less accommodation contributing to generation of fasciculations, and then relatively increases by membrane depolarization as the disease progresses.

Additionally in our study using ROC curves we estimated the diagnostic utility of the MEM, that after multivariate analyses remained independently associated with survival in ALS patients. The c-statistic showed Superexcitability 7ms moderate predictive ability in contrary with the CMAP amplitude which is considered the more reliable predictive factor regarding the conventional NCS [15]. This fact increases the significance of our results towards the elucidation of the ALS pathology.

The clinical significance of our results is that Superexcitability 7ms can be used as a biomarker not only for survival but also for disease progression. It becomes even more important if we consider the latest developments in research on the treatment of ALS, where the dysfunction of K^+^ channels has become a therapeutic target with significant results, improving the symptoms resulting from it such as fasciculations, but also stabilizing the axonal parameters excitability [6]. But even more valuable is the therapeutic perspective for delaying disease progression targeting the initial disorder that results in disruption of potassium channel homeostasis, like cytoplasmic aggregation of the RNA-binding protein TDP-43, leading to atypical RNA splicing and subsequent loss of crucial axon-supporting proteins such as stathmin-2 in the peripheral nervous system [16]. In this therapeutic strategy, parameters of axonal excitability, such as Superxcitability 7ms, could be used as electrophysiological biomarkers for future clinical studies.

### Limitations

Our study has some limitations. Firstly, the number of patients is not great, but this was a prospective study with difficulties following up all the patients till the end point. Secondly, because of the characteristic disease course in ALS patients and the resulting clinical deterioration, we could not perform enough follow-up electrophysiological assessments to assess the course of declining in axonal excitability parameters. Further longitudinal studies with long term follow-up multiple excitability tests are needed to reveal better the time-course changes of these parameters [17].

## Supplementary Information

Below is the link to the electronic supplementary material.

Supplementary Material 1
