# Supplementary material for: Motor neuron axonal excitability changes in the clinical course of amyotrophic lateral sclerosis
Source: Neurol Sci. 2025 Aug 25;46(11):5847–53. doi: 10.1007/s10072-025-08443-w (PMC12537592; doi:10.1007/s10072-025-08443-w)
Supplement: Supplementary file 1 — Supplementary Material 1 [file 10072_2025_8443_MOESM1_ESM.docx]

**Supplement file**

**Table 1. Motor axonal excitability measurements result in patients with ALS and controls.**

| **Variable** | **ALS patients** | **Controls** | **p-value** |
| --- | --- | --- | --- |
|  | **n=56** | **n=24** |  |
|  | | | |
| Refractoriness 2ms, % (median, IQRs) | 68.45 (42.54-85.49) | 50.01 (34.87-71.63) | **0.031** |
| Refractoriness 2.5ms, % (median, IQRs) | 20.14 (3.80-36.52) | 13.74 (4.51-24.30) | 0.443 |
| RRP % (median, IQRs) | 2.98 (2.62-3.26) | 2.91 (2.63-3.02) | 0.338 |
| Superexcitability, % (median, IQRs) | -28.47 (-32.57, -22.03) | -25.13 (-28.10, -21.84) | 0.090 |
| Superexcitability 5ms, % (median, IQRs) | -28.52 (-32.69, -21.06) | -26.61 (-29.08, -23.28) | 0.301 |
| Superexcitability 7ms, % (median, IQRs) | -25.83 (-30.42, -21.35) | -21.06 (-25.54, -18.10) | **0.013** |
| Subexcitability, % (median, IQRs) | 10.87 (7.58-14.59) | 15.01 (10.58-18.35) | **0.006** |
| TEd (10-20ms), % (median, IQRs) | 70.93 (64.86-75.53) | 68.90 (64.61-72.03) | 0.345 |
| TEd (90-100ms), % (median, IQRs) | 50.56 (46.60-54.35) | 45.44 (42.36-47.83) | **<0.001** |
| TEh (10-20ms), % (median, IQRs) | -76.96 (-83.68, -71.60) | -78.58 (-83.75, -74.37) | 0.578 |
| TEh (90-100ms), % (median, IQRs) | -122.40 (-144.17, -102.22) | -123.27 (-147.32, -108.15) | 0.508 |
| S-R slope, % (median, IQRs) | 4.87 (3.79-5.97) | 4.53 (3.71-5.86) | 0.571 |
| SDTC,ms (median, IQRs) | 0.48 (0.41-0.56) | 0.48 (0.41-0.54) | 0.914 |
| CMAP, mV (mean, SD) | 8.09 (2.96) | 11.09 (3.53) | **<0.001** |
